# Drilling into RIP1 biology: what compounds are in your toolkit?

**DOI:** 10.1038/cddis.2015.254

**Published:** 2015-09-17

**Authors:** S B Berger, J Bertin, P J Gough

**Affiliations:** 1 Pattern Recognition Receptor Discovery Performance Unit, Immuno-Inflammation Therapeutic Area, GlaxoSmithKline, Collegeville, PA 19426, USA

RIP1 kinase is rapidly emerging as an exciting therapeutic target. Having been implicated in the regulation of a broad range of key inflammatory cellular processes including cell death, cytokine production and inflammasome assembly, inhibition of this pathway has the potential to have a significant impact across a wide range of inflammatory diseases.^1^ However, a fundamental and complete understanding of the consequences of inhibiting RIP1 kinase activity is integral to informing future clinical directions. RIP1 is quite challenging in this regard, as it has independent kinase and scaffolding functions, limiting the utility of knockdown and knockout systems to recapitulate the effects of inhibitors.^2, 3^ The recently published kinase-dead knock-in mice may help to further understand this biology, but recent evidence demonstrating that RIP1 inhibitors may have function beyond blocking kinase activity also limits their value. These limitations of genetic manipulation coupled with the emerging complex biology of RIP1 makes it imperative to identify high-quality tools with a thorough understanding of their pharmacology and limitations.

Necrostatin-1 (Nec-1) was the first inhibitor of RIP1 kinase activity identified and has been instrumental in developing our understanding of RIP1 biology.^4^ It is a ‘non-traditional' structural template and consequently has excellent selectivity against other kinases. However, Nec-1 is limited by its modest potency, off-target activities and poor metabolic stability.^5^ A second version of Nec-1, termed Nec-1s, shares the kinase selectivity of Nec-1 and has an improved off-target profile and metabolic stability, but still remains modestly potent.^5^ More recent efforts have identified other inhibitors of RIP1 kinase activity, but these are ‘traditional' hinge-binding templates and also inhibit a varying number of other kinases.^6, 7^ These off-target kinase activities limit the utility of these compounds as tools, and careful attention must be paid to the concentrations and doses of the inhibitor used when attributing their biological activity to inhibition of RIP1.

In our new paper in *Cell Death Discovery* we describe the identification of GSK'963 (Figure 1); a structurally distinct, ‘non-traditional' RIP1 kinase inhibitor.^8^ GSK'963 offers several distinct advantages over other RIP1 inhibitors described to date. It is highly potent and displays more than a 100-fold increase in activity over Nec-1 and Nec-1s in cell-based assays. Furthermore, like Nec-1 and Nec-1s, GSK'963 is ultra-selective displaying 10 000-fold selective for RIP1 over any of the 339 kinases tested, and is a chiral molecule that can be used in conjunction with its inactive enantiomer GSK'962 to confirm on-target effects. We believe that these attributes of GSK'963 make it a superior next-generation *in vitro* tool which can be used in combination with the other available RIP1 inhibitors to help unravel RIP1 biology.

Despite having a number of *in vitro* compounds available, much less focus has been placed on identifying suitable *in vivo* tools. In the literature, Nec-1 remains the most commonly used RIP1 inhibitor *in vivo*. However, as previously mentioned, Nec-1 is only modestly potent and metabolically unstable which translates into poor pharmacokinetic properties and only brief inhibition of RIP1 kinase. This fact is highlighted in our *Cell Death Discovery* publication, as administering Nec-1 at the most commonly used dose in the literature has no effect in the acute, RIP1 kinase-dependent, TNF shock model. The absence of efficacy lines up well with our measurements of Nec-1 compound levels and predictions of RIP1 inhibition and as such, we believe it is critical for future publications to report *in vivo* drug concentrations in order to allow for a better interpretation of the extent of RIP1 inhibition. Nec-1s certainly provides significantly improved inhibition of RIP1 activity *in vivo*, and recent data suggest that it may be formulated for extended RIP1 coverage.^9^ As demonstrated in our new publication, GSK'963 represents another option for acute *in vivo* experimentation, but we believe the field will also benefit from the identification of other tool molecules for chronic studies.

As *in vitro* and *in vivo* pre-clinical data continue to emerge around the role of RIP1 kinase in driving inflammation and pathology, it is essential that we have a comprehensive understanding around the biology to inform clinical development efforts. Hence, it is imperative that we use the right tools in the right ways to draw the correct conclusions. GSK'963 represents the most potent and selective tool currently available to the field and will be highly useful in untangling the contributions of RIP1 to inflammatory mechanisms. It is currently an extremely exciting time for the field, as our understanding of the basic science has the potential to rapidly translate into benefit for patients.

## Figures and Tables

**Figure 1:**
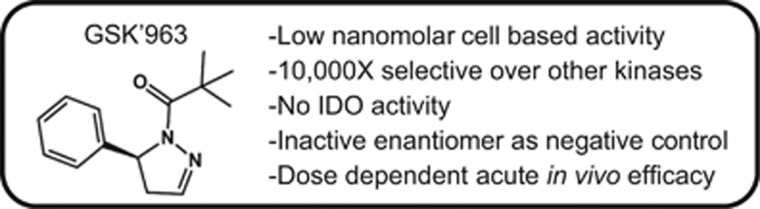
Newly identified RIP1 kinase inhibitor GSK'963. GSK'963 has many desirable properties compared to other commercially available tools, and should help to clarify our current understanding of the role of RIP1 in contributing to disease pathogenesis
